# Evaluation of a Rapid Immunochromatographic ODK-0901 Test for Detection of Pneumococcal Antigen in Middle Ear Fluids and Nasopharyngeal Secretions

**DOI:** 10.1371/journal.pone.0033620

**Published:** 2012-03-20

**Authors:** Muneki Hotomi, Akihisa Togawa, Shin Takei, Gen Sugita, Rinya Sugita, Masamitsu Kono, Yutaka Fujimaki, Yosuke Kamide, Akihiro Uchizono, Keiko Kanesada, Shoichi Sawada, Naohiro Okitsu, Yumi Tanaka, Yoko Saijo, Noboru Yamanaka

**Affiliations:** 1 Department of Otolaryngology-Head and Neck Surgery, Wakayama Medical University, Wakayama, Japan; 2 Sugita ENT Clinic, Chiba, Japan; 3 Fujimaki ENT Clinic, Chiba, Japan; 4 Kamide ENT Clinic, Shizuoka, Japan; 5 Sendai ENT Clinic, Kagoshima, Japan; 6 Nonohana ENT Clinic, Yamaguchi, Japan; 7 Sawada ENT Clinic, Kochi, Japan; 8 Department of Otolaryngology, Tohoku Rosai Hospital, Miyagi, Japan; 9 Otsuka Pharmaceutical Co., Ltd., Tokyo, Japan; Instituto Butantan, Brazil

## Abstract

Since the incidence of penicillin-resistant *Streptococcus pneumoniae* has been increasing at an astonishing rate throughout the world, the need for accurate and rapid identification of pneumococci has become increasingly important to determine the appropriate antimicrobial treatment. We have evaluated an immunochromatographic test (ODK-0901) that detects pneumococcal antigens using 264 middle ear fluids (MEFs) and 268 nasopharyngeal secretions (NPSs). A sample was defined to contain *S. pneumoniae* when optochin and bile sensitive alpha hemolytic streptococcal colonies were isolated by culture. The sensitivity and specificity of the ODK-0901 test were 81.4% and 80.5%, respectively, for MEFs from patients with acute otitis media (AOM). In addition, the sensitivity and specificity were 75.2% and 88.8%, respectively, for NPSs from patients with acute rhinosinusitis. The ODK-0901 test may provide a rapid and highly sensitive evaluation of the presence of *S. pneumoniae* and thus may be a promising method of identifying pneumococci in MEFs and NPSs.

## Introduction

Acute otitis media (AOM) is a common bacterial infection during childhood and frequently accompanies acute rhinosinusitis. By 3 years of ages, 50–70% of children have experienced at least one episodes of AOM, with 15–20% of children suffering from recurrent episodes of AOM [1], [2]. *Streptococcus pneumoniae* is the major bacterial cause of both AOM and acute rhinosinusitis, followed by *Haemophilus influenzae* and *Moraxella catarrhalis* [3]. Simultaneous cultures from middle ear fluids (MEFs) and nasopharyngeal secretions (NPSs) demonstrate the presence of identical pathogens [4].

Antibiotics are prescribed frequently for initial treatment of these infectious diseases. However, the choice of appropriate antibiotics is difficult since there is no rapid and accurate diagnostic test to identify the pathogen in the middle ear cavity. As a result, selective pressure by the frequent use of empirical antimicrobial treatment has increased the incidence of antibiotic-resistant pathogens in childhood [5]–[8]. One alternative to antibacterial therapy for physicians would be to observe the affected children without administering antibiotics [9], [10]. However, this alternative is undoubtedly accompanied by the risk of worsening infections [11], [12]. A safer approach to the treatment of AOM and acute rhinosinusitis would be the accurate and rapid determination of the causative pathogen such as *S. pneumoniae* followed by prompt antibacterial therapy using antibiotics appropriate for the detected pathogen. Identification of the causative pathogen as early as possible and selection of a suitable antibacterial drug are thus desirable to prevent persistent and recurrent infections. Unfortunately, an effective tool for the rapid diagnosis of middle ear infections had not yet been developed.

While bacterial culture with Gram stain is the gold standard for identifying *S. pneumoniae*, bacterial culture requires several days to complete [13]–[15]. In addition, the prior administration of antimicrobial agents reduces the ability of conventional bacterial cultures to accurately identify the pathogen. An antigen detection test can demonstrate the presence of non-viable bacteria. The latex agglutination test and countercurrent immunoelectrophoresis have both been applied to *S. pneumoniae* identification, but both are insufficient as routine diagnostic methods in general practice because of their lack of sensitivity and specificity [16]–[19]. At present, polymerase chain reaction (PCR) is considered the most sensitive and specific test, but it is both expensive and requires a complicated process [20].

The rapid immunochromatograpy test for the urinary pneumococcal antigen, the Binax NOW® *Streptococcus pneumoniae* test (Binax, Inc., Portland, MA), shows high specificity (>90%) and high sensitivity (50–80%) in adult pneumonia and is thus a useful tool for identifying severe pneumococcal pneumonia in adults [21]–[23]. However, the use of a urine sample as a diagnostic tool for AOM and acute rhinosinusitis has limited value, especially for children. Most children with pneumococcal AOM have antigen-negative urine samples [24]. In addition, the majority of healthy children carrying *S. pneumoniae* in the nasopharynx may have antigen-positive urine samples [25]–[28]. In children who recently received pneumococcal vaccination, the urinary test may be positive [29]. Sampling of urine of infants with AOM or rhinosinusitis may be inconvenient. The Binax NOW® urinary antigen test and the RAPIRUN® *S.pneumoniae* antigen detection test for sputum have been adapted for respiratory infections [23], [30]–[33].

The ODK-0901 test (Otsuka Pharmaceutical Co., Ltd., Tokyo, Japan) is a diagnostic kit that uses polyclonal antibodies to detect pneumococcal polysaccharides directly from MEFs and NPSs. The present study was designed to evaluate the ability of ODK-0901 test to detect *S. pneumoniae* among patients with AOM and acute rhinosinusitis in a clinical setting.

## Materials and Methods

### Study populations

This case-control study was conducted between December 2009 to March 2010 in seven hospitals and clinics in Japan. Patients with AOM and/or acute rhinosinusitis were eligible to be enrolled into this study without regard to age and gender, previous or current antimicrobial treatments, and inpatient or outpatient status. Diagnostic criteria for AOM included an acute onset of symptoms including ear pain, fever, and, for young children, crying combined with abnormal tympanic membrane findings with redness, bulging, and obliteration of landmarks. Specimens of MEFs were collected with the ATOMS® tap middle ear aspirator (LUMENIS Co., Ltd., Tokyo, Japan) or sterile swabs after myringotomy under anesthesia of the tympanic membrane. The diagnostic criteria for acute rhinosinusitis included acute onset of symptoms including nasal discharge, headache/irritability, and moist cough combined with postnasal discharge. Specimens of NPSs were obtained with the ATOMS® tap or sterile swabs. Immediately after testing via the ODK-0901 test and plating the specimens for cultures, the samples were stored at −80°C until real-time PCR was performed.

This study was approved by the Institutional Review Board of the Ethical Committee of Nishinomiya Kyoritsu Neurosurgical Hospital and Tohoku Rosai Hospital. Before collecting samples, informed consent was obtained from the patients or from the parents or guardians for pediatric patients.

### Bacterial cultures

Bacterial culture was used as the gold standard for the presence of *S. pneumoniae* in samples. Approximately 10 µl of sample collected by sterile swabs was plated on blood and chocolate agar plates and then incubated for 24 to 48 h at 37°C under a 5% CO_2_ environment according to standard laboratory procedures. *S. pneumoniae* were identified by alpha-hemolysis and colony morphology on 5% sheep blood agar, Gram stain characteristics, optochin sensitivity, and bile solubility. *H. influenzae* were identified by growth on chocolate agar, colony morphology, Gram stain characteristics, and a growth requirement for X and V factors. *M. catarrhalis* were identified by colony morphology, Gram stain characteristics, and the biochemical reaction of butyrate esterase.

### Detection of pneumococcal antigen by the ODK-0901 test

The ODK-0901 test uses rapid immunochromatography to detect pneumococcal C-polysaccharides (teichoic acid) (C-ps (TA)) and capsular polysaccharides by rabbit anti-pneumococcal polysaccharide polyclonal antibody immobilized on a nitrocellulose membrane. After a 5-min extraction of the sample using the extraction reagent, approximately 0.2 ml (4–5 drops) of the extracted sample was applied to an additional reservoir cup and incubated for 15 min with the ODK-0901 test at room temperature.

If there are any pneumococcal C-ps (TA) and capsular polysaccharides in the sample, it forms an immune complex with gold-colloid-binding anti-pneumococcal polysaccharide polyclonal antibody during the development of the sample and, subsequently, produces a red line, which is complemented by anti-pneumococcal polysaccharide polyclonal antibody on the test line. The gold-colloid-labeled antibody not complemented on the test line presents a red line complemented by anti-rabbit IgG goat polyclonal antibody on the control line after it passed the test line. Therefore, the sample is determined to be positive for pneumococcal polysaccharide when two red lines appear and is determined to be negative when there is only a control line. If the control line does not appear, the sample should be retested. Although the test requires approximately 15 minutes, if the two test lines are observed within 15 minutes, the sample can be determined to be positive.

Extractions of the 13 pneumococcal strains including D39 strain serotype 2, TIGR4 strain serotype 4, EF3030 strain serotype 19F and 10 clinical isolates of serotype 3, 6A (2 strains), 6B (2 strains), 4, 19A, 19F, 23F (2 strains) from the nasopharynx of children with AOM were used as positive controls. As negative controls extractions of 10 non-pneumococcal bacteria including nontypeable *H. influenzae* (3 strains), *Moraxella catarrhalis* (3 strains), *Streptococcus pyogenes* (4 strains) were used in this study.

The ODK-0901 test showed cross-reactivity only *Streptococcus mitis* (1 strain) but not for other streptococcus species including *S. anginosus* (1 strain), *S. agalactiae* (1 strain), *S. constellatus* (2 strains), *S. equi* (1 strain), *S. intermedius* (2 strains), *S. oralis* (1 strain), and *S. sanguinis* (1 strain). The ODK-0901 test also recognized the purified LTA/TA, C-ps (the Statens Serum Institute, Copenhagen, Denmark) and typed purified pneumococcal capsular antigen, type 1, 2, 3, 4, 5, 8, 9N, 12F, 14, 17F, 19F, 20, 22F, 23F, 25, 6B, 10A, 11A, 7F, and 15B (the American Type Culture Collection, Manassas, VA) (manufacture data).

### Real-time PCR

The detection of pneumococci by real-time PCR was done in accordance with a previously reported assay procedure in Kitasato Otsuka Biomedical Assay Laboratories, Kanagawa, Japan [32].

Briefly, total genomic DNA was extracted by the QIAamp DNA Mini Kit (QIAGEN, Valencia, CA). The relative amount of pneumococcal DNA genome was quantified by real-time PCR using primers and the TaqMan probe established for the region of the *pspA* gene of *S. pneumoniae*. Real-time PCR was then proceeded on a thermal cycler ABI7700 or ABI7900 (Applied Biosystems, Foster City, CA). The nucleotide sequences of the primers were as follows: forward primer: 5′-CAAGTCTAGCCAGCGTCGCTAT-3′; reverse primer: 5′-GGGAGATTCTTCTGCTCTTACAAAAG-3′, 5′-GGGAGATTCTTCTGCTCTTACCAAAG-3′, and 5′-GGGAGATTCTTCTGCTCTTACAACAG-3′; and carboxyfluorescein-labeled probe: 5′-(FAM)-CTGAGACGCAACAAAACCAGCCCC-(TAMRA)-3′, 5′-(FAM)-CTGAGACGTAACAAAACCAGCCCC-(TAMRA)-3′ and 5′-(FAM)-CGAAGACGCAACAAAACCAGCCCC-(TAMRA)-3′. RNase free water and DNA extracted from the *S. pneumoniae* ATCC6303 strain were used for negative and positive controls, respectively. After an initial denaturation at 95°C for 15 min, the PCR reaction was followed by 50 cycles of amplification at 94°C for 15 sec and at 60°C for 1 min. Positive and negative controls were included for every PCR run. The standard curves depended on the cycle threshold (Ct) values of the positive controls. The number of copies of the *pspA* gene in the samples was calculated based on the standard curve. The sample with less than 40 copies was defined as negative for real-time PCR.

### Statistics

Statistical analysis was done by Prism 5 (GraphPad Software, Inc., CA). A two tailed chi-square test or Fisher's exact test was used for categorical variables to test the significance of differences between groups. The pneumococcal DNA densities were compared by Mann-Whitney U test. A *p*-value of ≤0.05 was considered statistically significant. A 95% confidential interval (CI) was calculated.

## Results

### Populations

The 264 patients with AOM (250 with simple AOM and 14 with intractable OM) were enrolled in this study ranged in age from 0 to 56 years with a median age of 1 year and included 117 females and 147 males. There were 257 children (0 to 14 years old) and 7 adults (18 to 56 years old). The population of 268 patients with acute rhinosinusitis enrolled in this study included 264 patients with acute rhinosinusitis and 4 patients with acute exacerbation of chronic sinusitis and ranged in age from 0 to 75 years with a median age of 1 year. They were 249 children (0 to 14 years old) and 19 adults (18 to 75 year old). There were 124 females and 144 males. Two hundred and four patients had AOM and acute rhinosinusitis concurrently. Because of the small numbers of adult samples, we analyzed both MEF and NPS samples without regarding ages of patients. Finally, we obtained 264 MEFs including 14 otorrhea samples from patients with AOM and 268 NPSs from patients with acute rhinosinusitis.

### Bacterial cultures

When the samples were tested via conventional bacterial cultures, *S. pneumoniae* was identified in 59 samples out of 264 MEFs (22.3%). Of these 59 samples, 51 *S. pneumoniae* strains were a single pathogen and 8 *S. pneumoniae* strains were combined with other pathogens. Seven MEFs contained *S. pneumoniae* combined with either or both *H. influenzae* and *M. catarrhalis* (6 MEFs contained *H. influenzae* and oneMEF contained both *H. influenzae* and *M. catarrhalis*). *H. influenzae* and *M. catarrhalis* were identified in 84 (31.8%) and 9 (3.4%) MEFs, respectively. In 96 (36.4%) MEFs, no pathogenic bacteria were identified.

Out of 268 NPSs, *S. pneumoniae* was detected in 161 (60.1%) samples. Twenty-six NPSs contained *S. pneumoniae* as a single pathogen. In contrast to the MEF samples, for the NPS samples 134 strains were combined with either or both *H. influenzae* or *M. catarrhalis* (41 NPSs contained *H. influenzae*, 36 NPSs contained *M. catarrhalis* and 57 contained both pathogens). Only one strain was combined with other pathogenic bacteria. *H. influenzae* and *M. catarrhalis* were identified in 159 (59.3%) and 139 (51.9%) NPSs, respectively. No pathogenic bacteria were identified in 16 NPSs.

### Sensitivity and specificity of the ODK-0901 test for MEFs and NPSs

When the samples were evaluated with the ODK-0901 test, the pneumococcal antigen was detected in 88 (33.3%) MEFs. Compared with results obtained by conventional bacterial culture, the sensitivity, specificity, positive predicting value, and negative predicting value of the ODK-0901 test for MEFs were 81.4% (95% CI: 71.4%–91.2%), 80.5% (95% CI: 75.0%–85.9%), 54.5% (95% CI: 44.1%–64.9%), and 93.8% (95% CI: 90.1%–97.3%), respectively (Table 1).

**Table 1:** Sensitivity and specificity of the ODK-0901 test for MEFs and NPSs.

		Culture for NPSs			Culture for MEFs		
		Positive	Negative	Total	Positive	Negative	Total
ODK-0901	Positive	48	40	88	121	12	133
	Negative	11	165	176	40	95	135
	Total	59	205	264	161	107	268

On the other hand, the pneumococcal antigen was detected in 133 (49.6%) NPSs. The sensitivity was 75.2% (95% CI: 68.4%–81.8%), the specificity was 88.8% (95% CI: 82.8%–94.7%), and the positive and negative predicting values were 91.0% (95% CI: 86.1%–95.8%) and 70.4% (95% CI: 62.6%–78.0%) for NPSs, respectively, when compared with the results of conventional bacterial cultures (Table 1).

### Influence of prior antimicrobial treatment on the ODK-0901 test

The sensitivity and specificity of the ODK-0901 test for MEFs of patients who had undergone prior antimicrobial treatment were further evaluated. We defined “prior antimicrobial treatment” as antimicrobial treatment within 4 weeks before the MEFs and NPSs were obtained. The sensitivity, specificity, positive predicting value, and negative predicting value of the ODK-0901 test on MEFs from patients with prior antimicrobial treatment compared with from patients without prior antimicrobial treatment were 80.0% (95% CI: 59.7%–100%), 82.6% (95% CI: 73.6%–91.5%), 50.0% (95% CI: 30.0%–70.0%), and 95.0% (95% CI: 89.4%–100%) compared with 81.8% (95% CI: 70.4%–93.2%), 79.4% (95% CI: 72.6%–86.2%), 56.3% (95% CI: 44.1%–68.4%), and 93.1% (95% CI: 88.4%–97.7%), respectively (Table 2). The sensitivity, specificity, positive predicting value, and negative predicting value of the ODK-0901 test on NPSs from patients with prior antimicrobial treatment compared with patients without prior antimicrobial treatment were 67.4% (95% CI: 53.8%–80.9%), 90.0% (95% CI: 79.2%–100%), 91.2% (95% CI: 81.6%–100%), and 64.3% (95% CI: 49.7%–78.7%) compared with 78.3% (95% CI: 70.7%–85.8%), 88.3% (95% CI: 81.1%–95.4%), 90.9% (95% CI: 85.2%–96.5%), and 73.1% (95% CI: 64.1%–82.1%), respectively (Table 3). There were no statistically significant differences between the sensitivity, specificity, positive predicting value, and negative predicting value of the MEFs and NPSs from patients with prior antimicrobial treatment and those from patients without prior antimicrobial treatment. However, a tendency was observed for the sensitivity of the ODK-0901 test to decrease for NPSs from patients with prior antimicrobial treatment.

**Table 2:** Sensitivity and specificity of the ODK-0901 test for MEFs depending on prior antimicrobial treatment.

		With prior treatment			Without prior treatment		
		Culture positive	Culture negative	total	Culture positive	Culture negative	Total
ODK-0901	Positive	12	12	24	36	28	64
	Negative	3	57	60	8	108	116
	Total	15	69	84	44	136	180

**Table 3:** Sensitivity and specificity of the ODK-0901 test for NPSs depending on prior antimicrobial treatment.

		With prior treatment			Without prior treatment		
		Culture positive	Culture negative	total	Culture positive	Culture negative	Total
ODK-0901	Positive	31	3	34	90	9	99
	Negative	15	27	42	25	68	93
	Total	46	30	76	115	77	192

### Quantification of the *pspA* gene in MEFs and NPSs by real-time PCR

**Figure 1:**
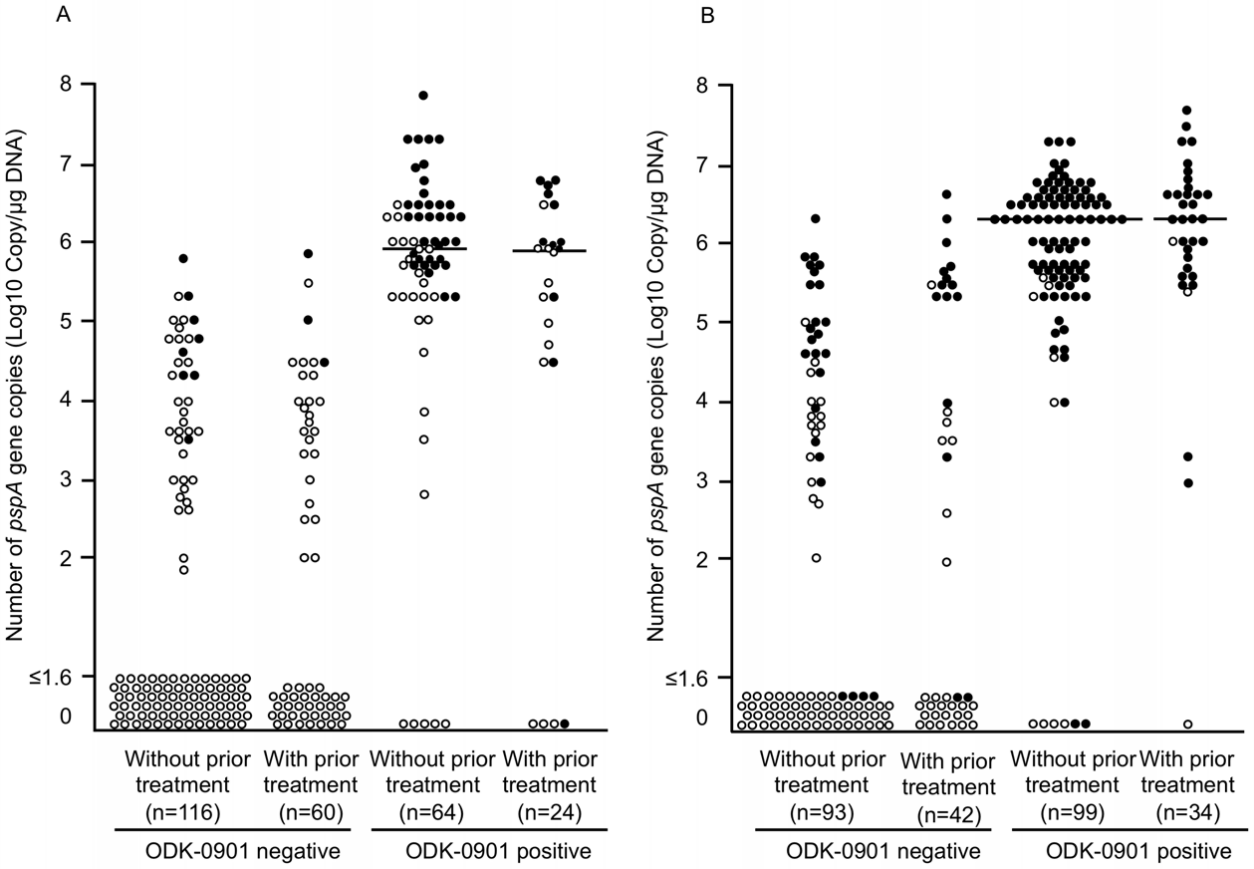
Distribution of the number of copies of *pspA* gene in MEF and NPS depending on prior antimicrobial treatment and the ODK-0901 test. The pneumococcal *pspA* gene was quantified by real-time PCR and the distribution was expressed. Open circles are culture-negative specimens. Closed circles are culture-positive specimens. A) Middle ear fluid; B) nasopharyngeal secretion.

The number of *pspA* gene copies in MEFs was significantly higher among samples positive for the ODK-0901 test than among those of samples negative for the ODK-0901 test (*p*<0.001) (Fig. 1A). The median number of *pspA* gene copies in ODK-0901-positive and -negative MEFs were 8.0×10^5^ copies/µg DNA and 40 copies/µg DNA, respectively. Sixty-four (36.4%) out of 176 MEFs negative for the ODK-0901 test contained the *pspA* gene, but 11 (6.3%) of them showed the growth of *S. pneumoniae* by the conventional culture. Nine (10.2%) out of 88 MEFs positive for the ODK-0901 test did not present the *pspA* gene. Only 1 (1.1%) MEF among the samples was positive by culture but not by the ODK-0901 test. There were no significant differences between the numbers of *pspA* gene copies in MEFs with or without prior antimicrobial treatment. The median number of *pspA* gene copies in ODK-0901-positive MEFs from patient having prior antimicrobial treatment compared with the median number from patients without prior antimicrobial treatment was 7.5×10^5^ copies/µg DNA versus 8.0×10^5^ copies/µg DNA.

The number of *pspA* gene copies in the ODK-0901-positive NPSs was also significantly higher than the number of copies in the ODK-0901-negative NPSs (*p*<0,001) (Fig. 1B). The median numbers of *pspA* gene copies in ODK-0901-positive and -negative NPSs were 2.0×10^6^ copies/µg DNA and 40 copies/µg DNA, respectively. Similar to the results from MEFs, the number of *pspA* gene copies in NPSs exhibiting growth of *S. pneumoniae* was significantly higher than the number of copies in NPSs negative for *S. pneumoniae* by culture (*p*<0.001). Fifty-six (41.5%) out of 135 ODK-0901-negative NPSs contained the *pspA* gene, and 34 (25.2%) of them were culture positive. Seven (5.3%) out of 133 NPSs positive for the ODK-0901 test did not have the *pspA* gene, but only 2 (1.5%) of them were culture positive. The median numbers of *pspA* gene copies in ODK-0901-positive NPSs from both patients having prior antimicrobial treatment and patients without prior antimicrobial treatment were 4.0×10^6^ copies/µg DNA.

### Predictive value for middle ear pneumococci by evaluating nasopharyngeal secretions

For 204 cases of AOM, we evaluated the ability of the ODK-0901 test on NPSs to accurately make a bacteriologic assessment of AOM when compared with the bacteriologic assessment resulting from conventional bacterial cultures. The positive and negative predictive values of nasopharyngeal conventional bacterial cultures to detect the presence of pneumococci in MEFs were 31.6% (95% CI: 23.6%–39.4%) and 100%, respectively. In contrast, the positive and negative predictive values of the ODK-0901 test to detect the presence of pneumococci in MEFs were 32.7% (95% CI: 23.8%–41.6%) and 92.8% (95% CI: 87.6%–97.9%), respectively (Table 4). There were no statistically significant differences between the abilities of conventional bacterial cultures and the ODK-0901 test to negatively predict middle ear pathogens.

**Table 4:** Prediction of middle ear pathogen by nasopharyngeal test.

			Middle ear fluid culture		
			Positive	Negative	Total
Nasopharyngeal secretion	Culture	Positive	42	91	133
		Negative	0	71	71
	ODK-0901	Positive	35	72	107
		Negative	7	90	97
	Total		42	162	204

## Discussion

Some attempts have been made to develop an immunochromatographic test suitable for the rapid detection of pneumococci in MEFs and NPSs in clinical situations. The advantage of such a test would be its ability to allow physicians to make earlier and more accurate decisions concerning the appropriate antimicrobial treatment for patients with AOM [24], [34]–[37]. In the present study, we evaluated the clinical significance of a novel immunochromatographic ODK-0901 test that would allow the rapid and accurate detection of pneumococci in MEFs and NPSs.

The ODK-0901 test works better than bacterial culture in detecting the presence of *S. pneumoniae* because it recognizes C-ps (TA) and capsular polysaccharides from *S. pneumoniae*, even though *S. pneumoniae* have died out by prior antimicrobial treatment or inappropriate culture conditions. Further, no cross-reactivity was seen with type b *H. influenzae* contained phosphorylcholine, suggesting that the antibody did not react with phosphorylcholine carried in C-ps [38]–[40].

In this study, conventional bacterial cultures showed that 22.3% of MEFs and 60.1% of NPSs contained viable *S. pneumoniae*. In contrast, the ODK-0901 test used for the study detected *S. pneumoniae* antigen in 33.3% of MEFs and 49.6% of NPS. The ODK-0901 test yielded 81.4% sensitivity and 80.5% specificity for MEFs and 75.2% sensitivity and 88.8% specificity for NPSs. Faden et al. first reported the application of the Binax NOW test for detecting *S. pneumoniae* in MEFs from otitis media with effusion (OME) with a sensitivity of 80.0% and a specificity of 83.0% [35]. On the other hands, Gisselesson-Solen et al. reported that the Binax NOW test had the relatively high sensitivity of 90.5% and specificity of 82.4% for severe AOM and associated complications [24]. While this study focused on simple AOM, it did compare the sensitivity and specificity of the ODK-0901 test with the previous results by the Binax NOW test. The ODK-0901 test can directly apply to MEFs and may prove useful in selecting the most appropriate therapy for AOM. In contrast, the sensitivity of ODK-0901 test for NPSs was low at 75.2%. The sensitivity and specificity of the Binax NOW test in previous reports varied from 92.2% to 95.0% and from 78.0% to 97.7%, respectively [34], [41].

Eleven samples out of 264 MEFs and 40 samples out of 268 NPSs exhibited false negatives to the ODK-0901 test due to the lower quantity of pneumococci in those MEFs and NPSs. Relatively small amounts of sample collected by swabs may lead to the false negative results for the ODK-0901 test. Some patients in this study with inconsistent ODK-0901 test results were concurrently undergoing or had undergone treatment with antibiotics. However, the study found that whether the patients had or had not undergone prior antimicrobial treatment made no differences in the pneumococcal density in MEFs and NPSs. As results, there were no differences in sensitivity and specificity of the ODK-0901 test based on the presence or absence of prior antimicrobial treatment. However, in particular, the ability of the ODK-0901 test to detect pneumococcal antigens in NPSs tended to be affected but not statistically significantly by the presence or absence of previous antibiotic treatment. Another possibility will be degeneration of polysaccharides of non-viable *S. pneumoniae* cells because real-time PCR indicated the presence of a relatively small amount of pneumococcal DNA.

In contrast, false positives were observed in 40 MEFs and 12 NPSs. Like the Binax NOW test, the ODK-0901 test has already been confirmed to exhibit cross-reactivity with *S. mitis*, part of the bacterial flora of pharynx (manufacture data) [42]–[44]. However, alpha-streptococcus species including *S. mitis* was not identified in the MEFs from the 40 false positive patients. Another possibility is that *S. pneumoniae* in MEFs are affected by various products of inflammation. Thus, the pathogen sometimes does not grow well in conventional culture tests and is thus very difficult to identify. The samples for the 75% of the MEFs and NPSs that were false positive for the ODK-0901 test but were found to contain pneumococcal DNA via real-time PCR may have been of such degraded quality that bacterial culture was not able to detect *S. pneumoniae*. The polysaccharide detection ability of ODK-0901 test as well as real-time PCR may be effective in *S. pneumoniae* with low biological activity. Furthermore, it is possible that some of the false positive samples contained a PspA null strain, which would result in the sample being negative for *pspA* gene via real-time PCR. Samples of 9 MEF and 7 NPSs were found to be negative by real-time PCR but were found to be positive by culture.

Since *S. pneumoniae* is one of the normal inhabitants of the nasopharynxes of children, its presence may create positives for antigen detection. Thus, the ODK-0901 test's detection of indigenous *S. pnuemoniae* in the nasopharynx will lead to overdiagnosis. In the current study, we further evaluated the pneumococcal DNA density in both MEFs and NPSs. The volume of MEFs obtained from AOM children is usually small and contains only a small number of organisms. It is important to evaluate the pneumococcal density in both types of specimens. Our study's use of real-time PCR proved that the ODK-0901 test yielded a positive result when the pneumococcal bacteria load was high at the affected site. Our previous study of nasopharyngeal carriage used real-time PCR to show that about 65% of children with upper respiratory infection had *S. pneumoniae* in the nasopharynx while conventional bacterial cultures of the same samples indicated that only 61% of the children were positive [45]. In practice, MEFs is not always available, and so nasopharyngeal secretions are sometimes used for bacteriological documentation. With the goal of using samples from nasopharyngeal colonization to predict the organism causing AOM, we evaluated the sensitivity, specificity, positive predictive value, and negative predictive value of culture test results from samples of middle ear fluid and nasopharyngeal secretions. We found essentially the same results as previous reports [24], [34], [35], [41]. It was reported that nasopharyngeal cultures has meaningful negative predicting value for determining middle ear pathogens [46], [47]. Based on the results of middle ear cultures as the gold standard, negative predicting value for *S. pnuemoniae* in MEFs were 100% by cultures and 92.8% by the ODK-0901 test, respectively.

The ODK-0901 test can thus be expected to be useful in infants from whom middle ear fluid cannot be collected. Because AOM may become persistent in young children, administration of appropriate antibiotics at an early stage of the treatment becomes especially important in both disease treatment and in the prevention of the development of drug-resistant bacteria. The current immunochromatography ODK-0901 test can become an important tool to help in the more rapid diagnosis of *S. pneumoniae* infections and in the subsequent administration of appropriate antibiotics earlier in the treatment cycle than was previously possible.
